# Surveillance for *Babesia odocoilei* in Hunter-Harvested Wild-Elk (*Cervus elaphus canadensis*) from Pennsylvania, USA (2016–2017)

**DOI:** 10.3390/vetsci8060094

**Published:** 2021-05-29

**Authors:** Elizabeth Jean Calvente, Clay Steber, Justin Brown, Holly Brown, Jeremiah Banfield, Nicole Chinnici

**Affiliations:** 1 Dr. Jane Huffman Wildlife Genetics Institute, 562 Independence Rd., Suite 114 East Stroudsburg University of Pennsylvania, East Stroudsburg, PA 18301, USA; ejcalvente@outlook.com (E.J.C.); csteber@ptd.net (C.S.); 2 Department of Veterinary and Biomedical Sciences, Penn State University, University Park, PA 16802, USA; jdb56@psu.edu; 3 VCA Metzger Animal Hospital, 1044 Benner Pike, State College, PA 16801, USA; hollymoorebrown@gmail.com; 4 Pennsylvania Game Commission, 2001 Elmerton Avenue, Harrisburg, PA 17001, USA; jebanfield@pa.gov

**Keywords:** *Babesia odocoilei*, Babesiosis, *Cervus canadensis*, elk, Pennsylvania, prevalence, surveillance, tick-borne disease, wildlife disease

## Abstract

*Babesia odocoilei* is a tick-borne protozoal parasite which infects the erythrocytes of members of the families Cervidae and Bovidae. Infection can result in hemolytic anemia, lethargy, anorexia, and death. The reservoir host of *B. odocoilei* is the white-tailed deer (*Odocoileus virginianus*); however, infections with overt disease have only been documented in reindeer (*Rangider tarandu tarandus*), caribou (*Rangider tarandu caribou*) and captive elk (*Cervus elaphus canadensis*). Infected elk may remain asymptomatic, creating the risk for dissemination of the pathogen when elk are relocated. Additionally, infected asymptomatic elk may contribute to the spread of *B. odocoilei* in the local wildlife/captive population via feeding ticks. Information regarding endemic regions of *B. odocoilei* infection is limited due to frequent asymptomatic infections and a lack of targeted surveillance of *B. odocoilei* in wildlife. To obtain data on *B. odocoilei* infection in wild elk in Pennsylvania, we tested blood samples collected from 190 hunter-harvested wild elk between 2016 and 2017. Of the 190 blood samples tested, 18.4% (35/190) tested positive for *Babesia* spp. Genetic sequencing of the positive samples showed a 98.0–100.0% match for *B. odocoilei*. No other *Babesia* species were identified. Results of this study documents *B. odocoilei* infection within hunter-harvested wild elk from Pennsylvania.

## 1. Introduction

Intraerythrocytic protozoal parasites of the genus *Babesia* are widespread throughout North America, infecting a variety of hosts. Potential for emerging diseases caused by the *Babesia* species is dependent on maintenance of the parasite via the cycle of transmission between competent vectors and vertebrate hosts within the environment. In North America, Ixodidae ticks are the only known vector for *Babesia* species, transmitting the parasite to vertebrate hosts while attached and feeding. High parasitemia of *Babesia* species in a host can lead to babesiosis, a disease with a range of symptoms dependent on the infecting species and host [1,2].

*Babesia odocoilei* falls within the informally named Small *Babesia* clade, Babesides, and is maintained in nature through a cycle involving white-tailed deer (*Odocoileus virginianus*) and blacklegged tick (*Ixodes scapularis*) [3,4,5,6,7]. Previously, infection was thought to be limited to members of the family Cervidae; however, recent publications have documented infection in desert bighorn sheep (*Ovis canadensis nelsoni*) and musk oxen (*Ovibos moschatus*), both within the family Bovidae [8]. Infections with *B. odocoilei* are often asymptomatic. Overt disease associated with infection has only been documented in reindeer (*Rangider tarandu tarandus*), caribou (*Rangider tarandus caribou*) and captive elk (*Cervus elaphus canadensis*), with symptoms emerging in naïve animals or from latency in a persistently infected animal [9]. Hemolysis and subsequent anemia are the primary results of babesiosis caused by parasite replication within erythrocytes and erythrophagocytosis via phagocytic cells [1,4]. Clinically infected cervids may present with mild anemia, acute hemolytic syndrome, or sudden death as the initial sign of infection [4,5,10].

*Babesia odocoilei* was previously thought to be restricted in the southwestern United States; however, recent publications show an expansion of occurrence in areas where blacklegged ticks are prevalent, including Pennsylvania, New York, and New Hampshire [8,11,12]. Climate change, changes in land use, and migration of ticks carried by animals (such as birds) have been suggested as some of the causes. Cases of infections in captive cervids and bovines have been reported in northeast United States and Canada, with recent cases of infected captive elk documented in New Hampshire and New York [5].

Acute babesiosis in elk is characterized by lethargy, hematuria, anorexia, and sudden death [13]. Fatal cases of *B. odocoilei* infection have been reported in captive elk and are typically associated with an outbreak within a captive population [9]. Subclinical, asymptomatic infection has been shown in captive elk, creating the potential for the animal to serve as an additional host for parasite transmission and maintenance in nature. Asymptomatic infection increases the likelihood of dissemination naturally through translocation of elk. Re-emergence of subclinical infections may occur during periods of stress such as rutting or calving season, causing elk to become symptomatic [9]. To the best of our knowledge, no documentation exists of clinical or subclinical babesiosis cases in wild elk.

To date, cases of clinical *B. odocoilei* infection in Pennsylvania have only been documented in captive reindeer [8]. The present study reports the prevalence of *B. odocoilei* in 190 hunter-harvested wild elk from Pennsylvania. To the best of our knowledge, this is the first reported study to investigate *B. odocoilei* infection in wild cervids and to document a high prevalence in wild elk.

## 2. Materials and Methods

### 2.1. Blood Collection and Storage

In 2016 and 2017, blood samples were collected during the 6-day hunting season in early November from wild elk harvested in five counties in Pennsylvania, USA: Elk county, Clearfield county, Cameron county, Centre county, and Clinton county. Prior to season, hunters were provided with supplies and instructions to collect a diversity of tissues and blood from their harvested elk. Immediately after harvest, hunters collected blood from the body cavity in two 15 mL conical polypropylene centrifuge tubes (Thermo Fisher Scientific™, Waltham, MA, USA). The blood samples were kept cool and delivered, along with the elk carcass, to the mandatory check station within 24 h of the animal being killed. At the check station, an aliquot of the blood was transferred to blood collection tubes containing calcium EDTA (BD Vacutainer^®^, Franklin Lakes, NJ, USA) and maintained at 4 °C. Within 24 h (< 48 h post-harvest), the tubes were delivered to the laboratory where they were frozen at −20° C until molecular testing was performed at the Dr. Jane Huffman Wildlife Genetics Institute (East Stroudsburg, PA, USA). 

### 2.2. DNA Extraction and PCR Identification

Using a Qiagen DNeasy® Blood and Tissue Kit (Qiagen Sciences Inc, Germantown, MD, USA), DNA was extracted from all blood samples following manufacturers protocol for non-nucleated blood. Pathogen identification was performed at the Dr. Jane Huffman Wildlife Genetics Institute. *Babesia* species screening was completed using a nested Polymerase Chain Reaction (PCR) protocol targeting the 18S rRNA gene. Forward and reverse primers used were BS1 (5’-GACGGTAGGGTATTGGCCT) and BS2 (5’-ATTCACCGGATCCACTCGATC) for external amplification, respectively, and the PiroA forward primer (5’-ATTACCCAATCCTGACACAGGG) and PiroC reverse primer (5’-CCAACAAAATAGAACCAAAGTCCTAC) for internal amplification [14]. Both external and internal amplifications were performed in a 20µL reaction consisting of 1X GoTaq Colorless Master Mix (Promega Corporation, Madison, WI, USA), dH2O, 1µM forward and reverse primer stock, and purified DNA. Thermal cycler conditions for external amplification were used with an initial denaturation of 94 °C for 5 min followed by 30 cycles of 94 °C for 1 min, 58 °C for 1 min, 72 °C for 90 s, with a final extension of 72 °C for 7 min. Thermal cycler conditions for internal amplification were used with an initial denaturation of 94 °C for 5 min followed by 30 cycles of 94 °C for 45 s, 57 °C for 45 s, 72 °C for 45 s, with a final extension of 72 °C for 7 min. Following PCR amplification, all PCR products were visualized using a 1.0% agarose gel stained with ethidium bromide. A post-PCR clean-up was performed on the second nested PCR product using ExoSAP-IT™ (Applied Biosystems, Foster City, CA, USA) and base pair sequences were generated using the BigDye™ Terminator v3.1 Cycle Sequencing Kit (Applied Biosystems, Foster City, CA, USA) and an Applied Biosystems 3130 genetic analyzer (Applied Biosystems Foster City, CA, USA). Sequence information was inserted into NCBI (National Center for Biotechnology information) Blast to evaluate PCR positive samples. All *Babesia* species positive blood samples were also screened for *B. microti* due to the prevalence in Pennsylvania, using a real-time PCR protocol targeting the 18S rRNA gene. Forward and reverse primers used were 5′-CAGGGAGGTAGTGACAAGAAATAACA-3′, 5′-GGTTTAGATTCCCATCATTCCAAT -3′and probe 5′-VIC TACAGGGCTTAAAGTCT MGBNFQ-3′ [15]. Amplification was performed in a 20µL reaction consisting of 1X Applied Biosystems™ TaqMan™ Fast Advanced Master Mix (Applied Biosystems, Foster City, CA, USA), dH_2_O, 1µM forward and reverse primer stock, and purified DNA. Thermal cycling conditions were used with 1 cycle of 50 °C for 2 min and 95 °C for 20 s followed by 45 cycles of 95 °C for 1 s and 60 °C for 20 s. A sample was considered positive if amplification passed a 0.733 threshold within 36 cycles. A synthetically produced DNA sequence of the *B*. *microti* 18S rRNA sample (Genewiz, South Plainfield, NJ, USA) was used to confirm accuracy of the assay and dH2O was used as a negative control.

### 2.3. Blood Smear Evaluation

Blood smears were prepared from samples collected during 2016 prior to freezing. Blood smears were stained with a Romanowsky-type stain using an automated slide stainer (Aerospray Hematology Stat Series 2, ELITechGroup, Puteaux, France) and examined under oil at 1000× magnification for the presence of intraerythrocytic protozoal organisms consistent with *Babesia* spp. [16]. Blood analysis was performed at VCA Metzger Animal Hospital (State College, PA, USA). 

### 2.4. Statistics

A chi-square analysis with Fisher’s exact test was performed to compare male to female ratios of elk positive for *B. odocoilei*. Statistical analysis was performed using IBM SPSS® Statistics for Windows version 24.0 (IBM, Armonk, NY, USA, 2016). The null hypothesis for each analysis assumed the compared groups would be equal and a significant difference indicated a measurable difference due to some factor other than chance. An alpha of 0.05 was used to determine statistical significance. 

## 3. Results

In total, 190 wild elk blood samples were collected during two hunting seasons in Pennsylvania: 85 from 2016 and 105 from 2017. None of the harvested elk were reported as appearing outwardly sick prior to harvest and no significant lesions were identified by the hunter during field dressing or at the check station. Through sequence analysis, a total of 18.4% (35/190) elk tested positive for *B*. *odocoilei*. Of the samples collected from the 2016 elk harvest, 17.4% (15/86) tested positive and from the 2017 samples, 19.2% (20/104) tested positive. Positive samples were confirmed as *B*. *odocoilei* via sequencing with a 98.0–100.0% match (GenBank Accession: MK986474.1). No other *Babesia* species were identified through sequencing, no samples tested positive for *B*. *microti* and no piroplasms were identified in the blood smears.

Proportion of *B. odocoilei* positive samples by county and sex are summarized in Table 1. No location or gender data were available for one elk in 2016 and one elk in 2017. These elk were not included in the totals presented in Table 1 and were negative for *B. odocoilei* and not included in the final analysis of positive blood samples. Using a Fisher’s exact test, the proportion of positive *B. odocoilei* elk was significantly higher among female than male elk (x^2^ = 5.881; *p* = 0.016). 

## 4. Discussion

In this study, we analyzed 190 blood samples collected from hunter-harvested wild elk in Pennsylvania (2016–2017). A total of 18.4% (35/190) of the elk tested were confirmed as positive for *B. odocoilei* via PCR/sequencing. Blood smears were negative for the presence of piroplasms and no other *Babesia* species were detected using molecular techniques. *Babesia odocoilei* was identified in all sampled counties and most were at relatively high prevalence (Table 1). To the best of our knowledge, this study is the first to document *B. odocoilei* in a wild Eastern elk population. Previous surveys for *B. odocoilei* focused on asymptomatic, captive elk herds on two farms in New Hampshire, USA, which found 100.0% *B. odocoilei* infections where the total number of elk tested was 32 [8]. Additionally, a farm in Indiana, USA found that 58.0% (34/59) of their elk population was infected with *B. odocoilei* [9].

Blood smears are not a specific diagnostic tool for identifying *Babesia* species due to the similarity of morphological characteristics to other blood-borne parasites such as *Theileria* [4]. Additionally, blood smears are not as sensitive as molecular techniques because the visualization of parasites in blood smears can be variable and is limited by the level of parasitemia. A study published in 2012 identified levels of parasitemia in symptomatic captive elk ranged between 5 and 20% of erythrocytes exhibiting protozoal parasites [13]. However, there are documented cases of acutely and peracutely infected, symptomatic elk that died from *B*. *odocoilei* but had a negative blood smear result; in these cases, *B*. *odocoilei* was confirmed via PCR testing [5]. Studies have documented blood smears to have a level of parasitemia detection between 0.01 and 0.1% for *B*. *ovis* in small ruminants and 0.001–0.1% for *Plasmodium* spp. in humans [17,18]. In comparison, the specificity and sensitivity of PCR allows for detection of much lower levels of parasitemia (0.0001–0.00001%) and more specific and accurate species identification [19,20,21]. An analysis of the use of PCR versus thin blood smears (TBSs) for detecting *B*. *microti* found the sensitivity (93% PCR vs. 84% TBS) and negative predictive value (83% PCR vs. 62% TBS) to be higher for PCR than TBS [22]. Overall, the level of parasitemia in asymptomatic hosts can be quite low, producing a negative result for animals that tested positive via PCR. The present study determined blood samples to be positive for *B*. *odocoilei* using molecular testing via PCR and sequencing. None of the blood smears examined in 2016 identified *B*. *odocoilei*. These results suggest that elk had subclinical, asymptomatic babesiosis with a low level of parasitemia, which could not be detected by using blood smears. Furthermore, these results support the use of molecular techniques as the gold standard for detection of parasitic infections such as *Babesia* in future surveillance studies for wildlife or captive populations.

Cases of clinical babesiosis in elk have only been documented in captive elk from zoos or elk farms [5]. Commonalities between these cases include a history of recent translocation of elk into captive populations, symptoms appearing during times of stress such as rutting, and follow up screening of the entire population leading to the discovery of subclinical, asymptomatic *B*. *odocoilei* infection within a portion of the remaining population [9,13,16,23]. The consensus in the published literature is that *B*. *odocoilei* infection in elk can remain latent until hosts experience periods of stress [9]. During these subclinical infections, it is possible that elk contribute to the spread of *B*. *odocoilei* in the local wildlife/captive population via feeding ticks [8,9].

There is an underrepresentation of infected female elk in documented cases of clinical babesiosis by *B*. *odocoilei* [5]. Rutting is a common stressor inducing infection from latency, resulting in more documented cases of male elk [5,8,9,13]. The present study found a higher proportion of *B*. *odocoilei* infection in female elk than males (Table 1). The present study found a significantly higher proportion of *B*. *odocoilei* infection in female elk than males. It should be noted that the Pennsylvania elk harvest is based on an allocated number of hunting tags, in which more female than male tags are issued, resulting in our sample size favoring female elk. Additionally, much of the published research on prevalence of *B*. *odocoilei* in elk is a response to a recent severe case of babesiosis in captive populations [5,9,13]. There is a lack of targeted surveillance of *B*. *odocoilei* in wildlife populations, limiting information regarding endemic regions of *B*. *odocoilei*. Although papers have been published with data of *B*. *odocoilei* positive ticks collected from environmental drags, the only location data specific to infected elk populations come from sporadic reports of captive elk following a case of severe babesiosis. As most of the literature is based on outbreaks of disease, the epidemiology and prevalence of infection in the absence of disease is unknown. Many of the authors publishing research in this field have suggested that cases of *B*. *odocoilei* infection in wildlife are possible where blacklegged tick populations are found, as they are the primary vector of the parasite. The same explanation has been suggested for the recent expansion of cases in the northeast into Canada of captive cervids with clinical and subclinical infections of babesiosis. Published Canadian reports have evaluated the possibility of migratory birds from the United States transporting infected blacklegged ticks with data suggesting this as a possible cause for the recent expansion of *B*. *odocoilei* into those regions [4].

Emergence of *B*. *odocoilei* infection throughout the northeast United States is thought to be related to the presence of the blacklegged ticks in this region [5,8,11]. The expansion of blacklegged ticks into new regions potentially due to climate change has created the possibility for dissemination of the pathogens harbored by this tick into new regions. Pak et al. (2019) published a 117-year retrospective analysis of Pennsylvania tick dynamics that found Elk county to have the highest population of blacklegged ticks [24]. Similarly, a two-year (2017–2018) tick surveillance study of the Pennsylvania elk population found Elk county to have the highest number of blacklegged ticks on elk for both years [25]. In the present study, Elk county had the highest proportion of *B*. *odocoilei* positive elk; however, bordering counties, Clearfield and Cameron, had relatively high prevalence of positive elk compared to counties not directly boarding Elk (Clinton and Centre). These data suggest a potential correlation between the high population of blacklegged tick in Elk county and the proportion of *B*. *odocoilei* positive samples in hunter-harvested elk in Pennsylvania. Additional surveillance is needed to further evaluate the association of blacklegged tick infestation and *B*. *odocoilei* infection in wild elk and other cervid species.

Although there is no current research indicating the ability of the winter tick (*Dermacentor albipictus*) to transmit *B*. *odocoilei*, many documented cases of clinical babesiosis have noted the presence of winter tick on infected elk [5,7,8,26]. In the present study, no tick data were collected in 2016; however, in 2017, two adult female elk positive for *B*. *odocoilei* from Centre and Clearfield counties had both winter ticks and blacklegged ticks present [25]. High infestations of winter ticks on Pennsylvania elk have been documented in Clearfield county causing dermatitis and potential mortality [27]. Additional studies are needed to evaluate vector competency of the winter tick for *B*. *odocoilei*.

The current Pennsylvania elk management plan for 2020–2025 indicates surveillance for tick-borne disease that does not include babesiosis [28]. To the best of our knowledge, this study is the first to document evidence of *B*. *odocoilei* infection in wild Pennsylvania elk. Continued surveillance of this protozoal parasite within the local tick and elk population is needed to better understand the risks of dissemination and clinical infection.

## 5. Conclusions

To the best of our knowledge, this study is the first to document the prevalence of *B*. *odocoilei* in a wild cervid population. The data herein expand on current *B*. *odocoilei* research that was previously limited by reports on captive populations only. Due to the absence of studies such as ours, endemic regions, epidemiology, and disease prevalence of *B*. *odocoilei* are unknown. Additionally, these results provide evidence of *B*. *odocoilei* infection in Pennsylvania wild elk. 

## Figures and Tables

**Table 1:** Demographic information on elk positive for *B.odocoilei*. Data separated by county and gender. No location or gender data were available for one elk in 2016 and one elk in 2017. Data below do not include these two elk.

Pennsylvania Elk Tested for *Babesia odocoilei*					
County Data			Gender Data		
County	Total Tested	Total (+)	Gender	Total Tested	Total (+)
Elk	97	19.6% (19/97)	Female	142	22.5% (32/142)
Clearfield	37	24.3% (9/37)			
Centre	26	7.7% (2/26)	Male	46	6.5% (3/46)
Cameron	18	27.8% (5/18)			
Clinton	10	0% (0/10)			

## Data Availability

The data presented in this study are available on request to the corresponding author.
